# Insight into the molecular mechanisms of gastric cancer stem cell in drug resistance of gastric cancer

**DOI:** 10.20517/cdr.2022.11

**Published:** 2022-07-01

**Authors:** Jixian Xiong, Tiantian Zhang, Penglin Lan, Shuhong Zhang, Li Fu

**Affiliations:** Guangdong Provincial Key Laboratory of Regional Immunity and Diseases, Department of Pharmacology and International Cancer Center, Shenzhen University Health Science Center, Shenzhen 518060, Guangdong, China.

**Keywords:** Gastric cancer, gastric cancer stem cells, drug resistance, chemotherapy, molecular mechanisms

## Abstract

Gastric cancer (GC) is one of the most common causes of cancer-related death worldwide, and gastric cancer stem cells (GCSCs) are considered as the major factor for resistance to conventional radio- and chemotherapy. Accumulating evidence in recent years implies that GCSCs regulate the drug resistance in GC through multiple mechanisms, including dormancy, drug trafficking, drug metabolism and targeting, apoptosis, DNA damage, epithelial-mesenchymal transition, and tumor microenvironment. In this review, we summarize current advancements regarding the relationship between GCSCs and drug resistance and evaluate the molecular bases of GCSCs in drug resistance.

## INTRODUCTION

According to GLOBOCAN estimates in 2020, gastric cancer (GC) is the fifth cause of global cancer incidence and the fourth leading cause of cancer mortality^[1]^. The incidence rates of GC vary widely across the world, with the highest rates in East Asia and Eastern Europe^[1]^. GC, most cases of which are gastric adenocarcinoma (GAC), is histologically divided into two subtypes [intestinal and diffuse (Lauren classification)]^[2]^ or four subtypes [papillary, tubular, mucinous, and poorly cohesive (WHO classification)]^[3]^. Based on genomic and epigenomic alterations, the most well-defined molecular-based classification systems include The Cancer Genome Atlas (TCGA) classification [EBV positive (EBV), microsatellite instable (MSI), genomically stable (GS), and chromosomal instable (CIN)]^[4]^ and the Asian Cancer Research Group (ACRG) classification [microsatellite instable (MSI), microsatellite stable TP53 inactive (MSS/TP53 inactive), MSS TP53 active (MSS/TP53 active), and MSS with epithelial–mesenchymal transition (EMT) features (MSS/EMT)]^[5]^. Despite advances in the field of early diagnosis in GC, most cases are still diagnosed at an advanced stage^[6]^ with unresectable or metastatic disease. Although current systemic treatments, including surgery, chemotherapy, radiotherapy, immunotherapy, and targeted therapy [Table 1] for advanced GC patients, have been considerably improved during recent decades, most patients with advanced GC die from tumor relapse and metastasis. The prognosis of advanced and metastatic GC remains poor, and the 5-year survival rate is < 10%^[7]^.

**Table 1:** A summary of systemic treatments in gastric cancer

**Treatment approaches**	**Mechanisms**	**Regimen**	**Efficacy**	**Adverse effects**
Surgery	Surgical resection	Open surgery, laparoscopic surgery, endoscopic resection, robotic surgery	Primary choice for early-stage GC	Low
Chemotherapy	Target and kill fast-dividing cells	Fluoropyrimidines, platinums, taxanes, irinotecan, *etc.*	The standard-of-care treatment for advanced GC	Damage to normal and healthy tissues
Radiotherapy	Ionizing radiation to target and kill tumor tissue	Ionizing radiation	Curative and palliative treatment	Damage to normal and healthy tissues
Targeted therapy	Target the specific molecules (HER2, VEGF, VEGFR, *etc*.)	Monoclonal antibodies and small molecule inhibitors	Use in combination with chemotherapy in first- and second-line settings	Relatively low
Immunotherapy	Block the binding of ligands to checkpoint receptors and re-activate the human cellular immune response	Immune checkpoint inhibitors (PD-1, PD-L1, and CTLA-4)	Use in second- and third-line settings	Relatively low

GC: Gastric cancer; HER2: human epidermal growth factor receptor 2; VEGF: vascular endothelial growth factor; VEGFR: vascular endothelial growth factor receptor; PD-1: programmed cell death protein 1; PD-L1: programmed cell death 1 ligand 1; CTLA-4: cytotoxic T-lymphocyte-associated protein 4.

Early GC patients can be cured with surgery alone. For advanced unresectable patients, chemotherapy represents the backbone of systemic therapy, and chemotherapy with or without radiotherapy has been integrated into standard-of-care therapies. Cytotoxic chemotherapy has been demonstrated to be effective for advanced GC patients, and the common cytotoxic chemotherapy drugs include fluoropyrimidines (e.g., fluorouracil, capecitabine, and S-1), platinums (e.g., cisplatin and oxaliplatin), taxanes (e.g., paclitaxel and docetaxel), topoisomerase inhibitors (e.g., irinotecan), and anthracyclines (e.g., doxorubicin and epirubicin). In patients with human epidermal growth factor 2 (HER2)-negative, advanced gastric adenocarcinoma, the current first-line treatment consists of two- or three-drug regimens. Doublet therapies are the combination of platinum derivatives (cisplatin and oxaliplatin) and fluoropyrimidine analogs (5-fluorouracil, capecitabine, and S-1). Three-drug regimens are the triplet combinations adding taxanes or anthracyclines (doxorubicin and epirubicin) to the doublet regimen. Table 2 summarizes the landmark trials for first-line treatment of advanced gastric cancer. However, the clinical benefit from these treatments is limited due to the toxicity of chemotherapeutic drugs and the development of drug resistance.

**Table 2:** Landmark trials in first-line treatment of advanced gastric cancer

**Studies**	**Treatment regimen**	**ORR**	**mPFS (mo), *P*-value**	**mOS (mo), ** ***P*-value**	**Reference**
MacDonald, *et al*. 1980	Fluorouracil, doxorubicin, mitomycin (FAM)	42%	NR	5.5	[8]
Wils, *et al.* 1991	Fluorouracil , doxorubicin, methotrexate (FAMTX) *vs*. FAM	41%/9%	NR	9.7/6.7	[9]
Webb, *et al.* 1997	Epirubicin, cisplatin, fluorouracil (ECF) *vs*. FAMTX	45%/21%	7.4/3.4, *P* = 0.00006	8.9/5.7, *P* = 0.0009	[10]
Van Cutsem, *et al.* 2006	Cisplatin, fluorouracil (CF) *vs. *Docetaxel,cisplatin, fluorouracil (DCF)	37%/25%	5.6/3.7, *P* < 0.001	8.2/9.6, *P* = 0.02	[11]
Cunningham, *et al.* 2008	Epirubicin, cisplatin, fluorouracil (ECF) *vs*. Epirubicin, cisplatin, capecitabine (ECX) *vs.* Epirubicin, oxaliplatin, fluorouracil (EOF) *vs.* Epirubicin, oxaliplatin, capecitabine (EOX)	41%/46%/42%/48%	6.2/6.7/6.5/7.0	9.9/9.9/9.3/11.2	[12]
Kang, *et al.* 2009	Cisplatin, capecitabine (XP) *vs.* Cisplatin, fluorouracil (FP)	41%/29%	5.6/5.0, *P* < 0.001	10.5/9.3, *P* = 0.008	[13]
Shah, *et al.* 2010	DCF, granulocyte stimulating factor (G-CSF) *vs.* modified DCF (mDCF)	33%/49%	6.5/9.7, *P* = 0.2	12.6/18.8, *P* = 0.007	[14]
Koizumi, *et al.* 2014	S-1, Docetaxel *vs.* S-1	38.8%/26.8%	5.3/4.2, *P* < 0.001	12.5/10.8, *P* = 0.032	[15]
Guimbaud, *et al.* 2014	epirubicin, cisplatin, capecitabine (ECX) *vs.* Folinic acid, Fluorouracil, Irinotecan (FOLFIRI)	39.2%/37.8%	5.3/5.8, *P* = 0.96	9.5/9.7, *P* = 0.95	[16]

ORR: Objective response rates; mPFS (mo): median progression-free survival (months); mOS (mo): median overall survival (months); NR: not reported.

More recently, targeted therapies have been developed for gastric cancer patients. These include trastuzumab, lapatinib, and margetuximab for epidermal growth factor receptor-2 (HER-2); bevacizumab for vascular endothelial growth factor (VEGF); ramucirumab, apatinib, and regorafenib for vascular endothelial growth factor receptor (VEGFR); cetuximab and panitumumab for endothelial growth factor receptor (EGFR); bemarituzumab for fibroblast growth factor receptor (FGFR); everolimus for mTOR; and zolbetuximab for Claudin 18.2. Various targeted therapy approaches have been investigated; however, several clinical trials in gastric cancer have failed due to the tumor heterogeneity and the difficulty of screening the beneficiary population for targeted therapeutic drugs. Furthermore, immunotherapy is being developed to block the binding of ligands to checkpoint receptors and re-activate the human cellular immune response. These are immune checkpoint inhibitors (ICIs) including nivolumab and pembrolizumab as PD-1 inhibitors, durvalumab and avelumab as PD-L1 inhibitors, and ipilimumab and tremelimumab as CTLA-4 inhibitors. Several trials have demonstrated that the benefits of immunotherapy only or with cytotoxic chemotherapy are relatively limited^[17]^. Thus, with relatively low response rates, the use of immunotherapy has only led to limited approval in the second-line treatment setting for GC^[18]^. More and more promising targeted therapies and immunotherapies are being investigated, and these will likely further improve outcomes for patients. Table 3 summarizes the landmark trials for targeted therapy and immunotherapy of advanced gastric cancer.

**Table 3:** Landmark trials in targeted therapy and immunotherapy [immune checkpoint inhibitors (ICIs)] for the treatment of advanced GC

**Items**	**Drugs**	**Treatment regimen**	**ORR, *P*-value**	**mPFS (mo), *P*-value**	**mOS (mo),** ***P*** **-value**	**Reference**
**Targeted therapies**	**HER-2**					
	Trastuzumab	Capecitabine or 5- FU plus cisplatin with Trastuzumab *vs. *Capecitabine or 5- FU plus cisplatin	47%/35%, *P* = 0.0017	6.7/5.5, *P* = 0.0002	13.8/11.1, *P* = 0.0046	[19]
	Trastuzumab	TrastuzumabDeruxtecan *vs. *Irinotecan or Paclitaxel	51%/14%, *P* < 0.001	5.6/3.5, *P* = 0.01	12.5/8.4	[20]
	Lapatinib	Paclitaxel with Lapatinib *vs. *Paclitaxel	27%/9%, *P* < 0.001	5.4/4.4, NS	11.0/8.9, NS	[21]
	Lapatinib	Capecitabine plus oxaliplatin with Lapatinib *vs. *Capecitabine plus oxaliplatin	53%/39%, *P* = 0.0031	6.0/5.4, *P* = 0.0381	12.2/10.5, NS	[22]
	Margetuximab	Margetuximab *vs.* Trastuzumab	25%/14%, *P* < 0.001	5.8/4.9, *P* = 0.03	21.6/19.8, NS	[23]
	**VEGF**					
	Bevacizumab	Capecitabine plus cisplatin with Bevacizumab *vs. *Capecitabine plus cisplatin	46%/37.4%, *P* = 0.0315	6.7/5.3, *P* = 0.0037	12.1/10.1, NS	[24]
	**VEGFR**					
	Ramucirumab	Ramucirumab *vs. *placebo	3%/3%, NS	2.1/1.3, *P* < 0.0001	5.3/3.8, *P* = 0.047	[25]
	Ramucirumab	Paclitaxel with Ramucirumab *vs. *Paclitaxel	28%/16%, *P* = 0.0001	4.4/2.9, *P* = 0.0001	9.6/7.4, *P* = 0.017	[26]
	Apatinib	Apatinib *vs. *placebo	2.84%/0%, NS	2.6/1.8, *P* < 0.001	6.5/4.7, *P* = 0.0149	[27]
	Regorafenib	Regorafenib *vs. *placebo	NR	2.6/0.9, *P* < 0.001	5.8/4.5, NS	[28]
	**EGFR**					
	Cetuximab	Capecitabine plus cisplatin with Cetuximab *vs. *Capecitabine plus cisplatin	30%/29%, NS	4.4/5.6, NS	9.4/10.7, NS	[29]
	Panitumumab	Epirubicin, oxaliplatin, and capecitabine with Panitumumab *vs.* Epirubicin, oxaliplatin, and capecitabine	46%/42%, NS	6.0/7.4, NS	8.8/11.3, *P* = 0.013	[30]
	**FGFR**					
	Bemarituzumab	Modified FOLFOX6 with Bemarituzumab *vs. *modified FOLFOX6	47%/33%	9.5/7.4, *P* = 0.073	Not reached/12.9, *P* = 0.027	[31]
	**mTOR**					
	Everolimus	Everolimus *vs. *placebo	4.5%/2.1%	1.7/1.4, *P* < 0.001	5.4/4.3, NS	[32]
	**Claudin 18.2**					
	Zolbetuximab	EOX with Zolbetuximab *vs. *EOX	39.0%/25.0%, *P* = 0.034	7.5/5.3, *P* < 0.0005	13.0/8.3, *P* < 0.0005	[33]
**Immunotherapy-Immune Checkpoint Inhibitors (ICIs)**	**PD-1**					
	Nivolumab	Nivolumab *vs. *placebo	11.2%/0%, *P* = 0.0088	6.1/1.61, *P* < 0.0001	11.6/5.26, *P* < 0.0001	[34]
	Nivolumab	CTx (S-1 or capecitabine plus oxaliplatin) with Nivolumab *vs. *CTx (S-1 or capecitabine plus oxaliplatin)	57.5%/47.8%, *P* = 0.0088	10.45/8.34, *P *= 0.0007	17.45/17.15, NS	[35]
	Pembrolizumab	Pembrolizumab *vs. *paclitaxel	16%/14%	1.5/4.1, *P* = 0.0007	9.1/8.3, *P* = 0.0421	[36]
	**PD-L1**					
	Durvalumab	Durvalumab plus tremelimumab, 2 L *vs.* Durvalumab, 2 L *vs.* Tremelimumab, 2 L *vs.* Durvalumab, tremelimumab, 3 L *vs.* Durvalumab, tremelimumab, 2 L/3 L	7.4%/0%/8.3%/4.0%	1.8/1.6/1.7/1.8	3.4/3.2/7.7/10.6,	[37]
	Avelumab	Avelumab *vs.* chemotherapy (paclitaxel or irinotecan)	2.2%/4.3%	1.4/2.7, NS	4.6/5.0, NS	[38]
	**CTLA-4**					
	Ipilimumab	Ipilimumab *vs.* first-line chemotherapy	1.8%/7.0%	2.72/4.90, *P* = 0.034	12.7/12.1	[39]
	Tremelimumab	Durvalumab plus tremelimumab, 2 L *vs.* Durvalumab, 2 L *vs.* Tremelimumab, 2 L & Durvalumab, tremelimumab, 3 L *vs.* Durvalumab, tremelimumab, 2 L/3 L	7.4%/0%/8.3%/4.0%	1.8/1.6/1.7/1.8	3.4/3.2/7.7/10.6,	[37]

ORR: Objective response rates; mPFS (mo) : median progression-free survival (months); mOS (mo) : median overall survival (months); HER-2: epidermal growth factor receptor-2; VEGF: vascular endothelial growth factor; VEGFR: vascular endothelial growth factor receptor; EGFR: endothelial growth factor receptor; FGFR: fibroblast growth factor receptor; mTOR: mammalian target of rapamycin; PD-1: programmed cell death protein 1; PD-L1: programmed cell death 1 ligand 1; CTLA-4: cytotoxic T-lymphocyte-associated protein 4; NS: no significant difference; NR: not reported.

**Figure 1:**
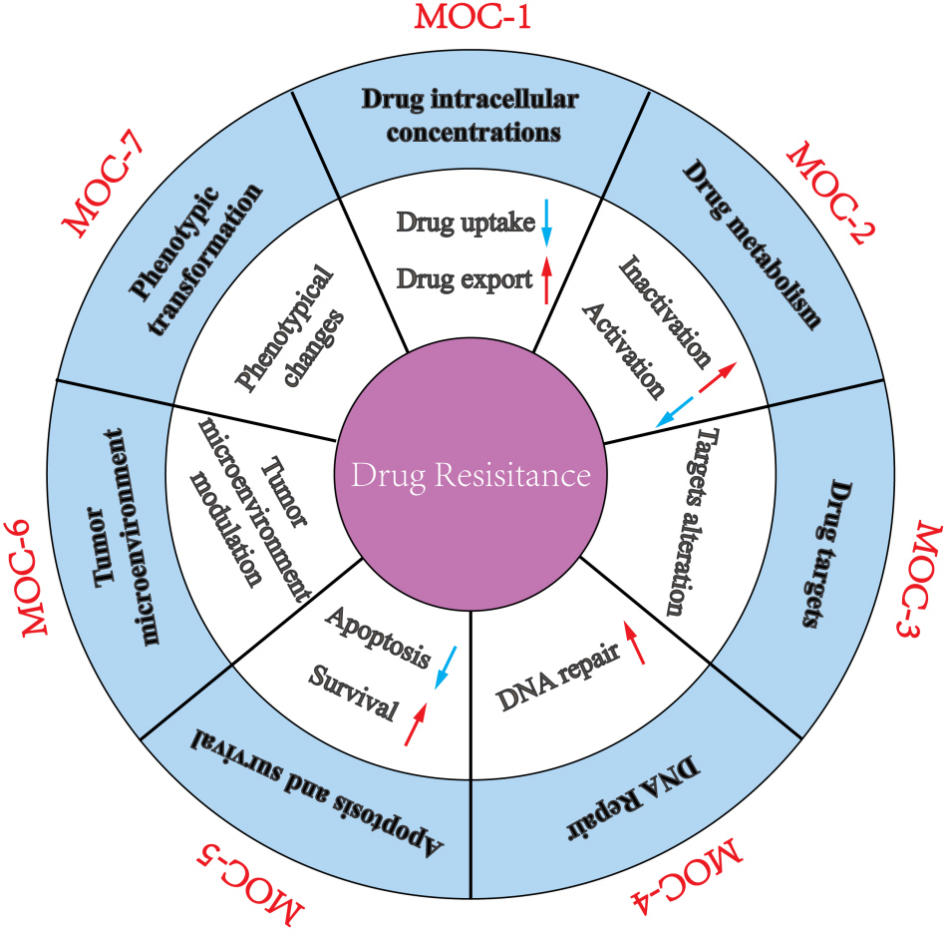
A schematic diagram depicting the molecular mechanisms accounting for drug resistance in gastric cancer.

**Figure 2:**
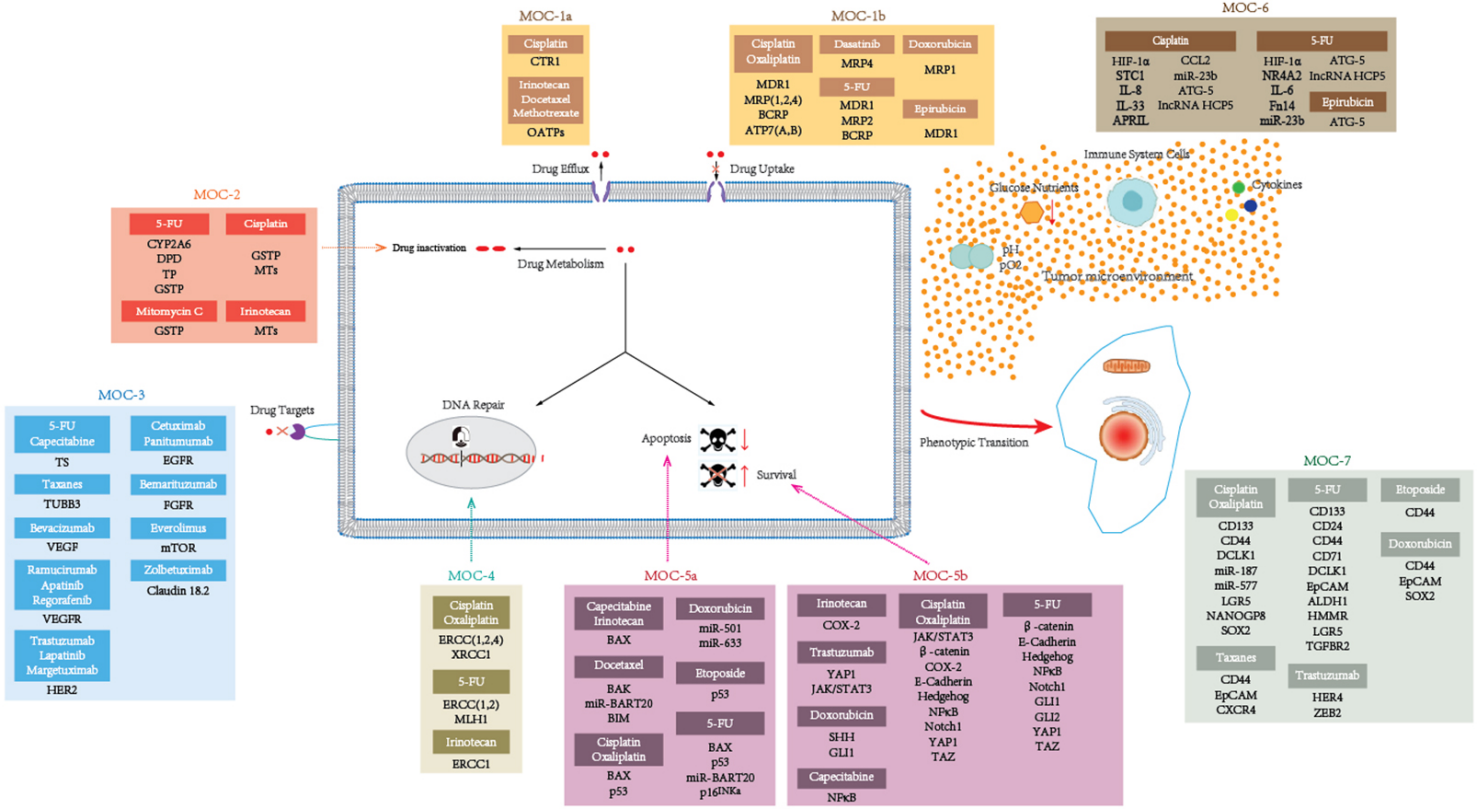
Proteins and non-coding RNAs accounting for drug resistance in gastric cancer. This figure is based on the work of Marin *et al*.^[40]^.

Drug resistance leads to pharmacological treatment failure and poor outcomes for advanced GC patients. The mechanisms of drug resistance of GC are divided into seven groups, according to the previously proposed classification^[40]^: change in drug intracellular concentration (MOC-1), change in drug metabolism (MOC-2), change in drug targets (MOC-3), change in DNA repair (MOC-4), change in apoptosis and survival (MOC-5), change in tumor cell microenvironment (MOC-6), and phenotypic transformation (MOC-7) [Figure 1]. We summarize the updated knowledge of the molecular mechanisms attributed to drug resistance in GC in Figure 2.

Cancer stem cells (CSCs) are a subpopulation of stem cell-like cancer cells that are responsible for cancer pathogenesis including initiation, development, drug resistance, metastasis, and cancer recurrence^[41-43]^. In recent years, accumulating evidence indicates the presence of CSCs in various types of cancers, including brain^[44]^, breast cancer^[45]^, head and neck cancer^[46]^, renal cancer^[47]^, colon cancer ^[48-50]^, pancreatic cancer^[51-52]^, liver cancer^[53]^, lung cancer^[54]^, prostate cancer^[55]^, and melanoma^[56]^, and targeting CSCs may be essential to prevent tumor relapse and spread^[57]^. Moreover, growing evidence suggests that there are several signaling pathways preferentially associated with CSCs^[58-60]^, including Hedgehog, Notch, WNT/β-catenin, JAK/STAT, PI3K/PTEN, and NF-κB pathways, which contribute to the survival, self-renewal, and differentiation properties of CSCs^[61]^.

CSC-targeting therapies are currently being investigated to reverse chemoresistance, including chemotherapeutic and biological agents that target stemness pathways including Hedgehog, Notch, Hippo/YAP1, JAK/STAT, and Wnt/β-catenin pathways; cancer stem cell surface markers including CD24, CD44, CD54, CD71, CD90, CD133, ALDH, CXCR4, EpCAM, LGR5, Sox2, and Oct4; the CSC microenvironment; and others^[62-64]^. However, these current strategies to target CSCs are not specific to CSCs, leading to toxic effects on normal tissues.

CSCs in GC were first identified from a panel of human GC cell lines^[65]^. Cancer stem cells from either human GC cell lines or tumor tissues were isolated using cell surface markers such as CD24, CD44, CD54, CD71, CD90, CD133, Lgr5, ALDH1, EpCAM, and CXCR4^[63,66-67]^. Although studies suggest the presence of gastric cancer stem cells (GCSCs), the origin of GCSCs is currently unclear and controversial. Two major hypotheses propose that GCSCs are derived from normal gastric stem cells (GSCs) or from bone marrow-derived mesenchymal stem cells (BM-MSCs)^[68,69]^.

In recent years, growing evidence shows that GCSCs play important roles in drug resistance in GC. Thus, understanding GCSC functions and their roles in drug resistance, as well as defining the molecular mechanisms of drug resistance, will help identify potential anticancer drug targets and develop new chemotherapeutic drugs and effective therapeutic strategies to improve the clinical outcomes of GC patients. In this review, we summarize our current understanding of the roles of GCSCs in GC drug resistance, as well as provide a comprehensive analysis of the potential molecular mechanisms by which CSCs contribute to drug resistance in GC.

## GCSCs AND DRUG RESISTANCE

Substantial studies have demonstrated that GCSCs are resistant to conventional radio-/chemotherapy. Aldehyde dehydrogenase (ALDH) is generally highly expressed in stem cells and considered as a CSC marker^[70]^. Gastric cancer cells with high expression of ALDH showed strong resistance to 5-fluorouracil (FU) and cisplatin; thus, high expression of ALDH in GC cell lines is believed to play a key role in resistance to chemotherapeutic drugs in GC^[71,72]^. Similarly, upregulation of LGR5, another GCSC marker, significantly enhanced cell stemness and drug resistance in MGC803 cells^[73]^. Further studies have shown that LGR5+ GCSCs are resistant to cisplatin treatment^[74]^. We recently showed that CD44+/CD54+ GCSCs isolated from cancer tissues can survive and expand after treatment with 5-FU and cisplatin^[75]^. Consistent with our results, another study showed that KHDRBS3 plays an important role in the acquisition of 5-FU resistance by regulating CD44 variant expression^[76]^. These results show that GCSCs play a key role in the acquisition of drug resistance in GC.

Accumulating evidence suggests the drug resistance capability of GCSCs is significantly higher than that of GC cells, and GCSCs can be enriched in GC after chemotherapy. Compared with GC cells, GCSCs showed stronger resistance to chemotherapeutic drugs 5-FU and oxaliplatin^[77]^. CSCs can be isolated or enriched by CSC-specific surface markers or through stem cell side population (SP) analysis^[78]^. Similarly, GCSCs isolated from GC cell lines by the SP method showed more resistance to chemotherapy^[79]^. Further study showed that CD44+ GCSCs isolated from tumor tissues were significantly enriched after treatment with 5-FU^[80]^. Another study demonstrated that ALDH+ CSCs in GC cell cultures can be enriched after treatment with cisplatin and 5-fluorouracil^[81]^. Meanwhile, clinical studies have revealed that resistance to anticancer drugs of GC is mainly associated with GCSCs. Patients with high CD133 expression exhibited stronger drug resistance, higher relapse rate, and lower five-year survival rate compared with patients with low CD133 expression^[82]^. Similarly, patients with high CD44 and CD133 expression showed worse survival^[83]^. Furthermore, expression of LGR5 and CD133 was identified to be significantly associated with poor clinical outcomes, and patients who are LGR5+ and CD133+ showed a lower overall survival rate than those who are LGR5- and CD133-^[84]^. The results from a phase II clinical trial show that GC patients with high expression CD44 who received chemotherapy with vismodegib, a hedgehog inhibitor, held a survival advantage^[85]^. Therefore, GCSCs are a major factor in GC resistance to radiation and chemotherapy.

## THE UNDERLYING MECHANISMS FOR GCSCS REGULATING THE DRUG RESISTANCE

**Figure 3:**
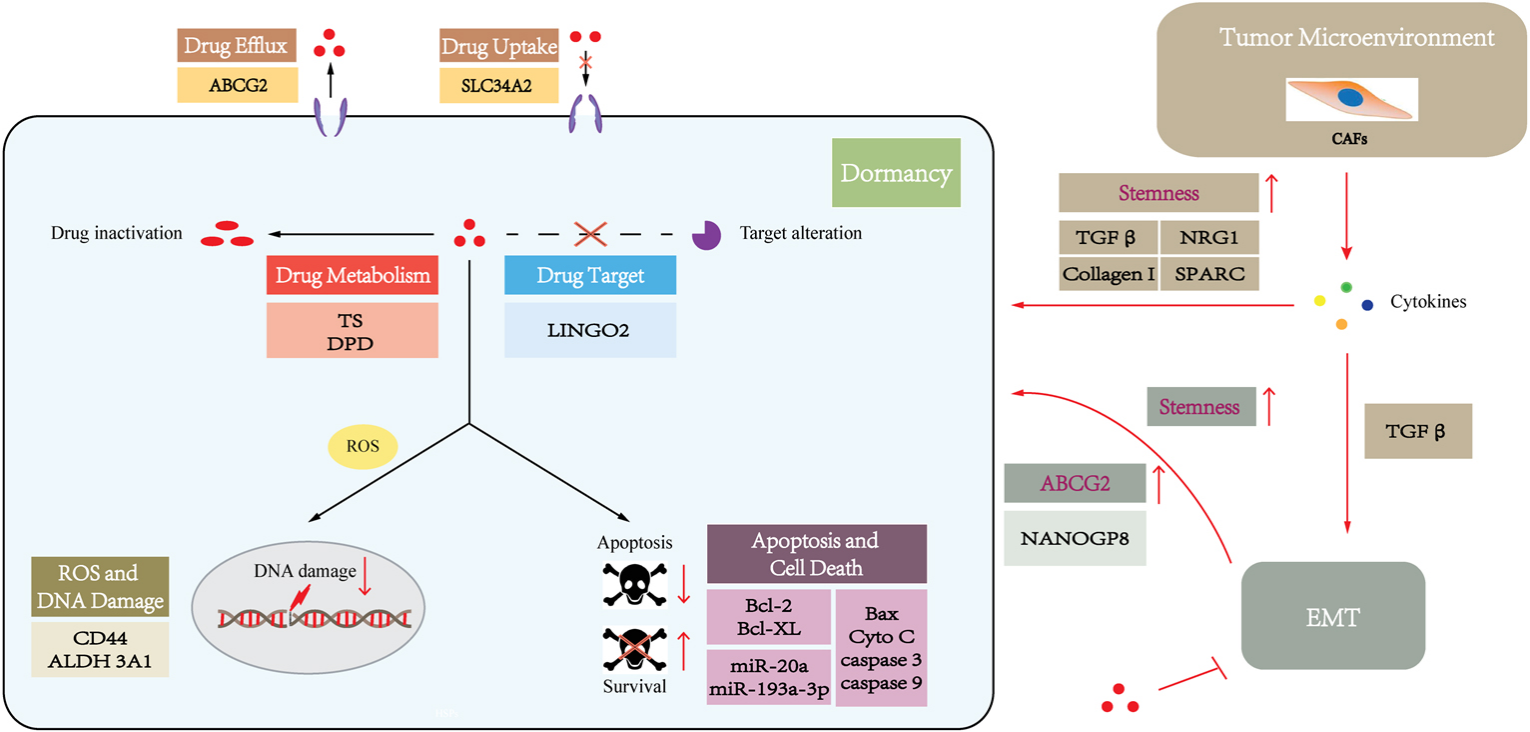
A schematic diagram depicting the molecular mechanisms accounting for chemoresistance in GCSCs. This figure is based on the work of Marin *et al.*^[40]^.

Drug resistance is a multifactorial phenomenon involving various components and multiple interrelated pathways, which work together to contribute to the development of this phenomenon. Various CSC-associated signaling pathways and molecular mechanisms have been described as implicated in CSC drug resistance^[86]^. To our knowledge, the underlying molecular mechanisms by which GCSCs contribute to chemoresistance include dormancy, drug trafficking, drug metabolism and targeting, apoptosis and cell death, DNA damage, epithelial-mesenchymal transition (EMT), and tumor microenvironment. The molecular mechanisms attributed to drug resistance in GCSCs are described below based on the previously proposed classification (MOC-1-7)^[40]^, and a schematic outline is summarized in Figure 3.

### Dormancy

Tumor dormancy contributes to the development of chemoresistance, metastasis, and cancer recurrence. CSCs are frequently in a quiescent state in which CSCs can remain in the G0/G1 stage with a low proliferation rate^[87,88]^. As most conventional chemotherapeutic drugs target proliferating cells, quiescence properties support CSCs to become resistant to radio- and chemotherapy^[72,89-90]^. Accordingly, 5-FU-resistant GC cells with CSC features were found to be mainly quiescent cells, which remained in the G0/1 phase^[91]^. Similarly, IL-17 enhances the proliferative capacity of quiescent gastric stem cells^[92]^, potentially promoting these transformed GCSCs to be sensitive to chemotherapy.

### Changes in drug uptake, efflux, metabolism, and targeting

#### Reduced drug uptake

One of the most studied mechanisms of cancer drug resistance is reducing the uptake of drugs. The uptake of drugs into tumor cells can be active transportation mediated by the membrane transporters. Most of these membrane transporters are solute carrier (SLC) proteins, which play an essential role in drug uptake. The SLC protein family contains more than 300 proteins that mediate the absorption of multiple types of substrates, including amino acids, sugars, organic cations, anions, *etc*., as well as chemotherapeutic drugs^[93]^. Unexpectedly, SLC34A2 was found to be increased in CD44+ GCSCs, and suppression of SLC34A2 in GCSCs reduced the effects of chemoresistance^[94]^. This result is consistent with SLC34A2 potentially having an oncogenic role in GC cells^[95]^. However, the detailed molecular mechanism remains unclear, which requires further investigation.

#### Increased drug efflux

Another mechanism of drug resistance is associated with the increased efflux of cytotoxic drugs by active ATP-binding cassette (ABC) transporter proteins, which is known as “drug efflux”. Forty-eight ABC transporter members have been identified in humans and are divided into seven distinct subfamilies (ABCA-ABCG) with different functions. Only 13 ABC transporters (ABCA2/3, ABCB1/2/5, ABCC1/2/3/4/5/6/10, and ABCG2) have been directly associated with chemoresistance^[96]^. Three major ABC transporters, including P-glycoprotein (P-gp/MDR1/ABCB1), multidrug resistance-associated protein 1 (MRP1/ABCC1), and breast cancer resistance protein (BCRP/ABCG2), have been found in various drug-resistant cancer cell lines and tissues and studied extensively for their correlation to multidrug resistance (MDR)^[97]^. These ABC transporters can lower the intracellular drug concentration by pumping out chemotherapy-based agents, including vinblastine, vincristine, doxorubicin, daunorubicin, actinomycin-D, and taxanes^[98,99]^.

CSCs express a high number of ABC transporter proteins on their cell surface^[64,86]^, and ABC transporters in CSCs have been shown to play an important role in drug resistance^[72,100-101]^. Therefore, ABC transporters are widely used as surface markers for CSC identification and isolation^[57]^.

ABCG2 is a major multidrug resistance pump, which is a downstream target of the sonic hedgehog (SHH)-glioma-associated oncogene homolog (GLI) signaling pathway^[102]^. Recent studies have indicated ABCG2 plays a pivotal role in drug resistance in GCSCs. CD44+/Musashi-1+ GCSCs with increased expression of ABCG2 exhibited resistant to doxorubicin^[103]^. Moreover, the inactivation of SHH-GLI signaling pathways decreased ABCG2 expression, rendering GCSCs more chemosensitive to doxorubicin^[103]^. This result implies that ABCG2 is a potential therapeutic target against CSCs to overcome drug resistance. Moreover, inhibition of ABCG2 expression by genistein, which is the predominant isoflavone in soy products, could inhibit gastric cancer stem cell-like features and reduce the chemoresistance of GCSCs^[104]^. Another study also demonstrated miR-132 could enhance cisplatin resistance in LGR5+ GCSCs via the SIRT1/CREB/ABCG2 signaling pathway^[74]^. However, the roles of other ABC transporter proteins of GCSCs in drug resistance are not clearly defined, and further investigations are needed to explore the roles of ABCs in cancer therapies against GCSCs.

#### Altered drug metabolism

Thymidylate synthase (TS) and dihydropyrimidine dehydrogenase (DPD) are both key 5-FU metabolic enzymes. TS is a target enzyme of 5-FU, and 5-FU exerts an anticancer effect by its conversion into fluorodeoxyuridine monophosphate (FdUMP) which can form a ternary complex with TS to cause suppression of the de novo synthesis of dTMP. DPD is the initial and rate-limiting enzyme that translates 5-FU into metabolites without cytotoxicity. TS and DPD, which are representative markers of 5-FU resistance, were shown to be significantly upregulated in a 5-FU-resistant CSC-like cell population in GC^[76]^. Hence, metabolic inactivation or alteration of the anticancer drugs in GCSCs enable CSCs to resist therapy and strengthen their stemness.

#### Changed drug targeting

Drug targeting altering by changing the expression and function of drug targets is also one of the major causes of drug resistance. Receptors of tyrosine kinase and its downstream signaling pathway play a pivotal role in carcinogenesis and tumor development and constitute the targets for tyrosine kinase inhibitors (TKIs). For instance, the EGFR signaling pathway is involved in the pathogenesis and progression of cancers by activation of either RAS/RAF/MEK/ERK or PI3K/AKT/mTOR cascade^[105]^. The activation of MEK, a component of the EGFR/Ras/RAF/MEK/ERK signaling pathway, caused drug resistance to MEK inhibitors^[106]^. Correspondingly, silencing LINGO2, a GCSC-related marker, reduced AKT, ERK, and MEK phosphorylation^[107]^, suggesting that activation of AKT, ERK, and MEK in GCSCs is responsible for chemoresistance to the inhibitors targeting these kinases.

### Inhibition of apoptosis and cell death

One of the primary goals of most anticancer agents is to cause tumor-selective cell death. The resistance to apoptosis, one of the key regulatory events leading to cell death, is the hallmark of cancer. Apoptosis occurs through extrinsic and intrinsic pathways that are dependent on caspase activation and mitochondrial outer membrane permeabilization (MOMP), respectively. The extrinsic apoptotic pathway is often related to ligands such as TNF-α, TNF-α-related apoptosis-inducing ligand (TRAIL), and Fas-ligand (FasL) and cell death receptors such as TNFR, TRAILR, FasR, linker proteins, and caspases 3, 6, 7, and 8. The intrinsic pathway is triggered by mitochondrial membrane disturbance following various stimuli including DNA damage and radio-/chemotherapy. Pro-apoptotic proteins such as Bax and Bak, as well as anti-apoptotic proteins such as Bcl2 and Bcl-XL are involved. Both intrinsic and extrinsic pathways activate caspases and ultimately lead to cell apoptosis. Increasing evidence suggests that disruption of the apoptotic pathway impacts resistance to anticancer drugs in GCSCs. Pro-apoptotic proteins including Bax, cytochrome C, caspase 9a, cleaved caspase 3, and cleaved caspase 9 were observed to be downregulated, while anti-apoptotic proteins Bcl-2 and Bcl-XL were upregulated in CD44+ GC cells compared with CD44- GC cells^[108]^. Moreover, in CD44+ GC cells, inhibition of miR-193a-3p can induce apoptosis by activating the mitochondrial apoptotic pathway and enhance the chemotherapeutic response of cisplatin^[108]^. Thus, GCSCs can induce resistance to drug-mediated apoptosis by upregulation or activation of anti-apoptotic proteins or downregulation or mutation of pro-apoptotic proteins. Similarly, miR-20a could increase cisplatin resistance in GC cells via modulating the anti-apoptotic factors livin and survivin^[109,110]^, whereas miRNA-19b, -20a, and -92a are proven to promote GCSCs properties^[111]^. miR-20a may also be involved in the development of chemoresistance in GCSCs by modulating apoptosis through livin and survivin.

### Repair and prevention of DNA damage

The dynamic balance between DNA damage and repair depends on the type of injury and the activity of a variety of repair mechanisms: nucleotide-excision repair (NER), base-excision repair (BER), mismatch repair (MMR), non-homologous end-joining (NHEJ), and homologous recombination (HR) systems. DNA damage-inducing agents are among the most effective treatment regimens in clinical chemotherapy. However, GCSCs can be resistant to DNA damage by drug treatment-induced reactive oxygen species (ROS) scavenging. Gastrointestinal cancer cells with high CD44 expression exhibited an enhanced capacity for GSH synthesis, resulting in defense against ROS^[112]^. CSC marker ALDH can facilitate detoxification by scavenging of ROS, as well as by producing antioxidant compounds such as NADP^[113]^. Aldehyde dehydrogenase 3A1 was found to be upregulated in gastric cancer stem-like cells^[114]^. Moreover, in multiple GC cell lines and hematopoietic malignancies, ALDH is highly expressed in ROS-low cells, and ALDH-high/ROS-low cells may be cancer-initiating cells (CISs)^[115-117]^, which are also called CSCs. These data indicate that ALDH+ GCSCs can enhance the resistance to chemotherapy or radiochemotherapy by reducing the level of ROS and avoiding DNA damage.

### Epithelial-mesenchymal transition

Epithelial-mesenchymal transition (EMT) is a process of lineage transition whereby epithelial cells lose their epithelial traits and acquire mesenchymal cell phenotypes, with corresponding changes in cell morphology and expression of surface markers^[118]^. EMT facilitates tumor cell migration, invasion, metastasis, and drug resistance^[119]^. Several cytokines, chemokines, and growth factors can trigger EMT by activation of a group of EMT-inducing transcription factors (EMT-TFs) such as SNAIL, SLUG, ZEB1/2, and TWIST^[120]^. EMT is regulated by a wide, complex, interactive molecular network including exogenous inducers, intracellular regulatory miRNA, epigenetic modulators, and cellular signaling pathways including MAPK, ERK, PI3K, SMADs, and Wnt/β-catenin^[121]^.

EMT has been shown to regulate the acquisition of stemness in multiple cancer cells^[122]^ and promote CSC stemness and quiescence that increase drug resistance^[123]^. EMT could induce CSC characteristics that increase drug resistance through different mechanisms including the hedgehog, Wnt, Notch, and Musashi signaling pathways, as well as the epigenetic regulator Bmi1^[124,125]^.

EMT activation confers drug resistance in CSCs through other mechanisms, including promoting drug efflux by increased levels of ABC pumps or inhibition of cell apoptosis by elevated expression of anti-apoptotic proteins such as Bcl-XL^[123-124,126]^. Correspondingly, NANOGP8, one of the pseudogenes in the NANOG gene family, is identified to be the main regulator of GCSCs, which can promote EMT/stemness and enhance chemoresistance^[127]^. NANOGP8 may confer gastric cancer cells with chemoresistance by upregulation of ABCG2^[127]^. However, the exact molecular mechanisms responsible for EMT and the resulting drug resistance in GCSCs remain uncertain.

### Adaptation to tumor microenvironment

CSCs are found in a specialized tumor microenvironment (TME), known as the niche, which is mainly composed of extracellular matrix (ECM), cancer-associated fibroblasts (CAFs), cancer-associated adipocytes (CAAs), and endothelial, mesenchymal, and immune cells, and those conditions promote CSC adaptation^[128-129]^. Reciprocal interactions between CSCs and the niche are critical for CSCs to maintain their stemness properties and promote tumor initiation, metastasis, and drug resistance^[130]^.

Increasing evidence highlights that the TME takes part in therapeutic resistance in GCSCs, largely involving CAFs, which remarkably influence the TME via the secretion of various growth factors, cytokines, and chemokines^[131]^. The main component secreted by CAFs is TGFβ, which induces EMT^[132]^ and promotes the acquisition of GCSC features^[133]^, ultimately leading to drug resistance^[134]^. Another study showed that CAFs can also promote stemness by the secretion of NRG1, which activates the NF-κB signaling in GC^[135]^. Moreover, CAFs can induce drug resistance not only by promoting stem-related signaling pathways in GCSCs but also by secreting type I collagen, which contributes to decreasing drug uptake^[136]^. Additionally, a recent study demonstrated that low expression of gastric CAF-derived SPARC (secreted protein acidic and rich in cysteine) can promote GCSC transformation and 5-FU resistance^[137]^, suggesting that CAF-secreted SPARC may be involved in the regulation of drug resistance of GCSCs. Collectively, this evidence implicates an important role of the TME in the development of drug resistance of GC.

### Exosomes

Exosomes (about 30-200 nm) are small extracellular vesicles (EVs) that originate from endosomes and are secreted by live cells into the extracellular space through the fusion of multivesicular bodies (MVBs) with the plasma membrane^[138]^. They are composed of a transmembrane protein-containing lipid bilayer and cell-state-specific molecules including DNAs, mRNAs, ncRNAs, and proteins in the vesicle lumen. Exosomes, as carriers, mediate cell-to-cell communication and substance exchange via the transfer of donor cell-derived contents to recipient cells^[139]^. Increasing evidence suggests that tumor-derived exosomes play critical roles in many aspects of cancer, including tumor growth, metastasis, angiogenesis, immunity, and other processes, and can be used as potential diagnostic biomarkers or therapeutic targets for cancer patients^[140-142]^. Recent studies showed exosomes are associated with the transfer of the drug resistance phenotype, and cancer cells could develop drug resistance after the incorporation of exosomes from drug-resistant cancer cells. Studies indicated exosomal PD-L1 promotes chemoresistance via inducing T cell exhaustion, by which the T cells cannot be reinvigorated by anti-PD-1 treatment^[143]^. Inhibition of exosomal PD-L1 has also been reported to enhance the efficacy of anti-PD-L1 treatment^[143,144]^. Furthermore, another study showed chemotherapeutic agents stimulated the secretion of ABCB1-enriched exosomes from drug-resistant cells and increased the transfer of ABCB1 to the recipient cancer cells, thus assisting these sensitive cancer cells in developing the resistant phenotype^[145]^. More recently, it has been shown that exosomal transference of wild-type EGFR to EGFR-mutated sensitive cancer cells promotes resistance to the mutant-selective EGFR inhibitor osimertinib by activating the MAPK and PI3K/AKT signaling pathways^[146]^. Thus, exosomes could be novel therapeutic targets, which could overcome resistance to chemotherapeutic drugs^[105,106]^ or antibody-based approaches^[143,144]^, and they also might serve as a predictive biomarker for clinical responses to anti-PD-1 therapy^[144]^.

Increasing evidence also highlights that exosomes are involved in the drug resistance of CSCs. The underlying mechanisms are complex, including cell cycle blockage, increased drug efflux, upregulation of detoxifying enzymes, enhanced anti-apoptotic capacity and DNA repair efficiency, inducing EMT process, and immunosuppression^[147,148]^. However, the physiological and functional properties of exosomes in GCSCs are still unknown and need further investigation.

### Extrachromosomal circular DNA

Extrachromosomal circular DNA (eccDNA) refers to a type of double-stranded circular DNA that originates from but is independent of chromosomes, which is widely present in various eukaryotic cells and can be derived from anywhere in a genome with sizes ranging from hundreds of base pairs (bp) to several megabases (Mb)^[149]^. According to the size and origin, eccDNAs can be categorized into organelle eccDNAs such as mitochondrial DNAs (mtDNAs) or non-organelle eccDNA such as telomeric circle (t-circles), microDNA (100-400 bp), small polydispersed circular DNA (spcDNA) (100 bp-10 kb), episomes, and double minutes (DMs) (100 kb-3 Mb)^[150]^. eccDNAs play important roles in gene regulation, sponging of transcription factors, environmental adaptation and evolution, aging, immune response, cell-to-cell communications, and tumor development^[151,152]^.

**Figure 4:**
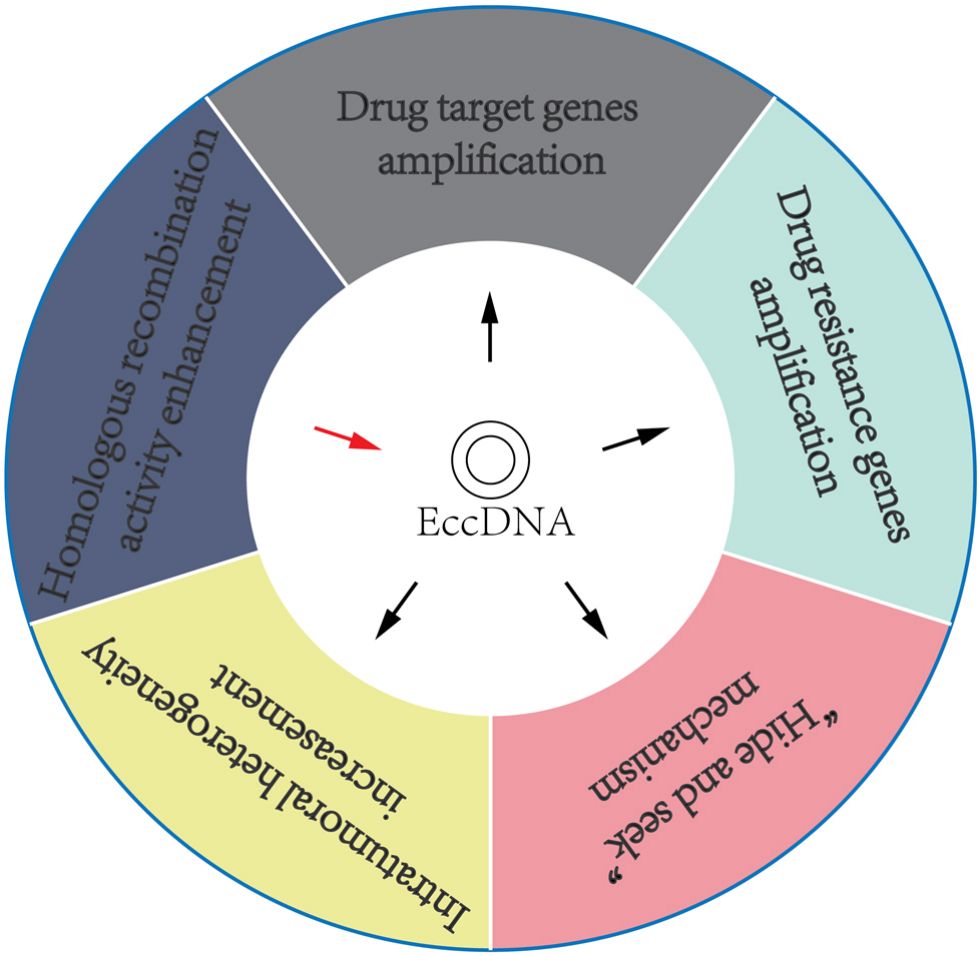
Overview of our current understanding of cancer drug resistance mechanisms induced by eccDNAs.

eccDNAs have been shown to help cancer cells develop drug resistance via various mechanisms [Figure 4]. (A) Amplification of drug target genes: For instance, DMs, which contain the gene coding for dihydrofolate reductase (DHFR), were identified to be amplified and associated with the development of methotrexate (MTX) resistance^[153]^. (B) Amplification of multidrug resistance (MDR) genes: DMs, bearing the multidrug resistance 1 (MDR1) gene, were amplified in human epidermoid carcinoma cells and caused resistance to various anticancer drugs by upregulation of MDR1^[154]^. (C) “Hide and seek” mechanism: EGFRvIII, an oncogenic variant, can induce tumor cells to be more sensitive to EGFR tyrosine kinase inhibitor (TKI). Previous studies have demonstrated that erlotinib resistance in glioblastoma is caused by the elimination of DMs containing EGFRvIII^[155,156]^. However, after erlotinib withdrawal, the mutant EGFR re-emerged on DMs, which induced GBM cells to be re-sensitive to erlotinib treatment^[156]^. Through this “hide and seek” mechanism, cancer cells can evade drug therapy by dynamic modulation of drug-targeted oncogenes residing on eccDNAs. (D) Increasing intratumoral heterogeneity: eccDNAs can drive heterogeneity among daughter tumor cells, thus inducing these cells to obtain survival advantage under drug pressure^[150]^. (E) Increasing homologous recombination activity: Homologous recombination is associated with eccDNA biogenesis. Recent studies have shown that homologous recombination activity was increased in DM-carrying MTX-resistant colon cancer cells, whereas inhibition of homologous recombination activity decreased the expression of DM-containing genes and enhanced drug sensitivity in MTX-resistant cells^[157]^.

eccDNAs contribute to a variety of features in cancers and may serve as novel, promising molecular markers to shed new insights into the diagnosis, prognosis, and treatment of cancer patients. However, the functions and underlying mechanisms of eccDNA in CSCs are still unclear and require further exploration.

### Helicobacter pylori infection

*Helicobacter pylori* (*H. pylori*) infection remains a main risk factor in the development of GC. In 1994, *H. pylori* was diagnosed as a Group I carcinogen by the WHO (World Health Organization)^[158]^. The stem cell hypothesis of cancer formation is that stem or progenitor cells can acquire CSC characteristics, evade homeostatic control, and lead to carcinogenesis. *H. pylori* has been shown to induce EMT and cancer stem cell (CSC)-like properties in gastric epithelial cells^[159,160]^ and gastric cancer cells^[161,162]^. Data from multiple studies show that *H. pylori* may directly interact with gastric stem/progenitor cells^[163-164]^ or bone marrow-derived cells (BMDCs)^[165]^ to impact the status and properties of these cells, which could be responsible for generating GCSCs. Moreover, *H. pylori* infection can induce inflammation, impact the local microenvironment, and affect gastric stem/progenitor cells and their differentiation by inducing genetic or epigenetic alterations^[166-168]^. *H. pylori *infection can mediate oncogenic transformation by inducing GCSCs generation or affecting gastric stem/progenitor cells. However, the underlying mechanisms leading to GCSC emergence and the resulting drug resistance in GCSCs in response to* H. pylori *infection are awaiting further investigation.

## CONCLUSION

Conventional radio-/chemotherapy provides a limited effect on prolonging the survival of advanced GC patients, and, recently, accumulating evidence shows that GCSCs are resistant to conventional chemotherapy and play a direct role in tumor metastasis and relapse. Based on the extensive evidence presented in this review, it is obvious that GCSCs regulate tumor radio-/chemoresistance via multiple intrinsic and extrinsic mechanisms. This review aims to provide an understanding of the precise mechanisms underlying GCSC resistance to chemotherapeutic drugs. Identifying the molecules and revealing insight into their interaction networks through further investigations may help to discover novel targets of anticancer therapy, develop new therapeutic approaches for the prevention of tumor recurrence and resistance, and increase the lifespan of GC patients.

However, to date, molecular mechanisms of drug resistance in GCSC remain largely unclear. Many aspects are still in need of further clarification: (1) to find more key components or molecules for regulating GCSCs resistance to the anticancer agents; (2) to define the precise molecular mechanisms and clarify how GCSCs coordinate these different, complex molecular pathways to response the chemotherapeutic drugs; and (3) to find the specific GCSC markers related to its response to the anticancer agents, so as to evaluate the effectiveness of different drugs and therapeutic strategies. More importantly, many more need to be proven to be effective in the clinic. We are at the beginning of understanding drug resistance from gastric cells to GCSCs. More basic and clinical studies should be done to increase the knowledge about the mechanisms of drug resistance to improve the outcome of advanced GC patients.

## DECLARATIONS

### Copyright

© The Author(s) 2022.
